# Enhanced Photocatalytic Degradation of Perfluorooctanoic Acid by Mesoporous Sb_2_O_3_/TiO_2_ Heterojunctions

**DOI:** 10.3389/fchem.2021.690520

**Published:** 2021-05-19

**Authors:** Xinyun Yao, Jiaqi Zuo, Yu-Jue Wang, Ning-Ning Song, Huang-Hao Li, Kaipei Qiu

**Affiliations:** ^1^ State Environmental Protection Key Laboratory of Environmental Risk Assessment and Control on Chemical Process, Shanghai, China; ^2^ School of Chemistry and Molecular Engineering, East China University of Science and Technology, Shanghai, China; ^3^ Shanghai Environmental Protection Key Laboratory for Environmental Standard and Risk Management of Chemical Pollutants, School of Resources and Environmental Engineering, East China University of Science and Technology, Shanghai, China; ^4^ China Environmental Protection Foundation, Beijing, China; ^5^ Shanghai Institute of Pollution Control and Ecological Security, Shanghai, China

**Keywords:** perfluorooctanoic acid, photocatalysis, TiO_2_, heterojunction, mesoporous

## Abstract

Perfluorooctanoic acid (PFOA), a typical perfluorinated carboxylic acid, is an emerging type of permanent organic pollutants that are regulated by the Stockholm Convention. The degradation of PFOA, however, is quite challenging largely due to the ultra-high stability of C-F bonds. Compared with other techniques, photocatalytic degradation offers the potential advantages of simple operation under mild conditions as well as exceptional decomposition and defluorination efficiency. Titanium dioxide (TiO_2_) is one of the most frequently used photocatalysts, but so far, the pristine nanosized TiO_2_ (e.g., the commercial P25) has been considered inefficient for PFOA degradation, since the photo-generated hydroxyl radicals from TiO_2_ are not able to directly attack C-F bonds. Mesoporous Sb_2_O_3_/TiO_2_ heterojunctions were therefore rationally designed in this work, of which the confined Sb_2_O_3_ nanoparticles in mesoporous TiO_2_ framework could not only tune the band structure and also increase the number of active sites for PFOA degradation. It was found that, after loading Sb_2_O_3_, the absorption of UV light was enhanced, indicating a higher efficiency of light utilization; while the band gap was reduced, which accelerated the separation of photo-generated charge carriers; and most importantly, the valence band edge of the Sb_2_O_3_/TiO_2_ heterojunction was significantly lifted so as to prevent the occurrence of hydroxyl radical pathway. Under the optimal ratio of Sb_2_O_3_–TiO_2_, the resulting catalysts managed to remove 81.7% PFOA in 2 h, with a degradation kinetics 4.2 times faster than the commercial P25. Scavenger tests and electron spin resonance spectra further revealed that such improvement was mainly attributed to the formation of superoxide radicals and photo-generated holes, in which the former drove the decarboxylation from C_7_F_15_COOH–C_7_F_15_^•^, and the latter promoted the direct electron transfer for the conversion of C_7_F_15_COO^−^–C_7_F_15_COO^•^. The Sb_2_O_3_/TiO_2_ photocatalysts were highly recyclable, with nearly 90% of the initial activity being retained after five consecutive cycles, guaranteeing the feasibility of long-term operation.

## Introduction

Perfluorooctanoic acid (PFOA) is an important industrial surfactant that used to be widely adopted in many applications (Wang Z. et al., 2017). As a typical perfluorinated carboxylic acid, the high stability of PFOA makes it extremely difficult to decompose (Wang S. et al., 2017), and its broad distribution in a variety of environments have been confirmed by a large number of studies (Giesy and Kannan, 2002; Post et al., 2012; Zareitalabad et al., 2012; Houtz et al., 2013; Ghisi et al., 2019), causing significant concerns on human health (Sunderland et al., 2018). Therefore, it has been listed in the Stockholm Convention as a persistent organic pollutant to be eliminated (UNEP, 2019). A wide range of removal techniques have been developed over the past decades, such as physical adsorption by powdered/granular activated carbon, anion-exchange resin, or biochar (McCleaf et al., 2017; Xiao et al., 2017; Gagliano et al., 2020; Park et al., 2020), and redox treatment triggered by the photochemical, sonochemical or electrochemical processes (Hori et al., 2004; Hori et al., 2005; Moriwaki et al., 2005; Lin et al., 2012; Trojanowicz et al., 2018). Among them, photocatalytic degradation allows an easy operation under mild conditions, and offers the possibility to fully decompose PFOA with high conversion efficiency.

Titanium dioxide (TiO_2_) is one of the most studied photocatalysts for environmental remediation, which upon the exposure of UV light, generates positive holes at its valence band to enable the rapid conversion of surface adsorbed water or OH^−^ into OH^•^ radicals for the subsequent pollutant oxidation (Nakata and Fujishima, 2012; Pelaez et al., 2012; Schneider et al., 2014; Nasr et al., 2018). However, such a photo-generated OH^•^ strategy has been found not feasible for the degradation of PFOA (Li et al., 2012), as the oxidation potential value of hydroxyl radicals (276 kJ/mol) is not enough to break either the C-F bond (ca. 460–540 kJ/mol) or C-C bond (347 kJ/mol), and is even inefficient to turn C_7_F_15_COO^−^ into C_7_F_15_COO^•^, which is often regarded as the rate-determining step in the oxidative degradation of PFOA (Wang S. et al., 2017). To facilitate the direct electron transfer from PFOA, early studies were conducted in an acidic (HClO_4_) solution, at a pH below the p*Ka* of PFOA (2.8), which enhanced the PFOA ionization to form C_7_F_15_COO^−^ (Dillert et al., 2007; Panchangam et al., 2009). On the other hand, it was also reported that the addition of oxalic acid as a hole-scavenger for photo-excited TiO_2_ led to the formation of a strong reductant, carboxyl anion radical (CO_2_^•−^), which were able to directly convert the molecular C_7_F_15_COOH into C_7_F_15_^•^ radicals (Wang and Zhang, 2011). Apart from the above, another versatile approach was to modify TiO_2_ with noble metals, non-noble metals, or metal-free carbon supports (Estrellan et al., 2010; Song et al., 2012; Chen et al., 2015; Chen et al., 2016; Li et al., 2016; Gomez-Ruiz et al., 2018), through which the photo-generated electrons were trapped, reducing the electron-hole recombination and prolonging the lifetime of holes.

Here, in this study, mesoporous Sb_2_O_3_/TiO_2_ heterojunctions were rationally designed to improve the photocatalytic degradation of PFOA. Incorporation of porous structures has been long regarded as an effective strategy to promote the performance of TiO_2_ photocatalysts (Du et al., 2011; Zhou et al., 2014), but surprisingly, it has not been applied for the removal of PFOA yet, as far as the authors are aware. Compared with pristine TiO_2_, the mesoporous one features a higher surface area/more active sites, as well as faster mass transfer. Nanosized Sb_2_O_3_ were further embedded into the mesoporous TiO_2_ framework via a facile hydrothermal method. Antimony trioxide is a novel semiconductor with a broad band gap over 3 eV, but the photocatalytic activity of pure Sb_2_O_3_ nanoparticles is fairly low (Karunakaran et al., 2010). Coupling Sb_2_O_3_ with another semiconductor, mainly the TiO_2_, has been explored previously (Li et al., 2001; Liu et al., 2012; Wang et al., 2018; Wang et al., 2019), but none of them has attempted to investigate the photocatalytic activity on PFOA degradation. In this regard, it is hypothesized that the combination of a mesoporous structure and the integration of Sb_2_O_3_/TiO_2_ heterojunctions may enhance the PFOA removal kinetics.

## Experimental

### Materials

All materials were of analytical grade and used as received. Antimony chloride (SbCl_3_) was purchased from Aladdin Industrial Corporation. Perfluorooctanoic acid (C_7_F_15_COOH, 96% purity), tetrabutyl titanate (C_16_H_36_O_4_Ti), and ethanol were obtained from Sigma-Aldrich. Deionized water (DI water) was used in all experiments.

### Synthesis of Photocatalysts

20 ml tetra-n-butyl titanate (TBOT) were added dropwise to 200 ml DI water, and afterward, the mixture was left for 36 h at room temperature. The samples were then filtered, washed with DI water and ethanol for 3 times, and dried in vacuum oven at 60°C for 12 h to obtain the mesoporous TiO_2_.

To synthesize Sb_2_O_3_/TiO_2_ composites, 0.028 g of SbCl_3_ was dissolved in 20 ml ethanol and 0.5 g mesoporous TiO_2_ were added into 20 ml DI water. Both of these two solutions were stirred for 20 min, then mixed together, and further stirred for 30 more min. The pH of the mixture was adjusted to neutral with ammonia. After that, the suspension was transferred to a 200 ml Teflon-lined autoclave and heated at 180°C for 10 h. After the autoclave was naturally cooled to room temperature, the resulting light blue sample was separated by centrifugation, washed with DI water, and dried at 60°C for 12 h. The final dry powder was labeled as 1%-Sb_2_O_3/_TiO_2_. Similarly, in order to prepare 3-, 7-, or 10%-Sb_2_O_3_/TiO_2_, the dosage of SbCl_3_ was adjusted to 0.089, 0.196, or 0.280 g, respectively, and the other procedures remained the same.

### Characterization

The crystallite structures of Sb_2_O_3_/TiO_2_ and TiO_2_ were characterized using X-ray powder diffraction (XRD). Spectra were collected on a D8 Advance diffractometer (Bruker D8 Discover, United States) using Cu-Kα radiation. Infrared absorption spectra were conducted on a NICOLET 380 Fourier transform infrared (FT-IR) spectrometer (NICOLET, America). The morphologies and structures of the samples were characterized by transmission electron microscopy (TEM, FEI Teanci G2 F20, United States) and scanning electron microscopy (SEM, Hitachi SU8220, Japan) operating at an accelerating voltage of 200 kV. X-ray photoelectron spectroscopy (XPS) data was obtained using a Escalab 250Xi spectrometer (Thermo Fisher Scientific, China) with monochromatic Al KR radiation. DR-UV of the powders was obtained by diluting with 60 wt% BaSO_4_, pressing into a wafer, and measured using a Shimadzu UV-2450 spectrometer. Pore volume and Brunauer-Emmett-Teller (BET) surface area measurements of the synthesized Sb_2_O_3_/TiO_2_ were analyzed through N_2_ adsorption/desorption isotherms with a Micromeritics Surface Area and Porosity Analyzer (Micromeritics ASAP 2020, United States). Photoluminescence (PL) emission spectra were measured with Picoquant Fluo Time 300 equipped with a Xenon lamp (λ = 250–800 nm). Time-resolved emission spectra (TRES) were collected after excitation with 280 and 375 nm lasers.

### Photocatalytic Procedure and Perfluorooctanoic Acid Analysis

The photocatalytic reactor was equipped with one 4W UVC Ushio G4T5 low pressure mercury germicidal lamps and a stir plate. In a typical photocatalytic PFOA degradation experiment, 5 mg of the photocatalyst (2.5 g/L Sb_2_O_3_/TiO_2_ or TiO_2_) was first dispersed into a 50 ml quartz round bottom flask containing 20 ml of DI water dissolved with 10 ppm PFOA. Initial pH for most experiments was without adjustment. At defined time interval, aliquots were extracted, and filtered with 0.22 µm syringe filters. The concentration of the remaining PFOA, during the photocatalytic activity test, was measured by V-630 UV-vis spectrometer (Jasco International Co., Japan) and ultra-high performance liquid chromatography-mass spectrometry system (UPLC-MS, Thermo Fisher Scientific TSQ Quantum). A C-18 column (ZORBAX eclipse XDB, 2.1, 100, and 3.5 mm, Agilent Technologies, United States) was used for chromatographic separation. The column temperature and the flow rate were set at 20°C and 150 μL/min, respectively. The mobile phase of eluent A (2.5 mmol/L ammonium acetate water solution) and eluent B (acetonitrile). The gradient of eluent B was started with 30%, increased to 70% at 4 min, then decreased to 30% at 7 min, and maintained for another 3 min to keep stabilization. The samples were analyzed by multiple reaction monitoring in negative ion mode. Calibration curves of PFOA was in the linear range from 0.1–20 ppm. The limit of detection was 1 ppb at the signal to noise ratio of 3.

Electron paramagnetic resonance (EPR) spectroscopy measurement was conducted by a spectrometer (JES FA300, Japan). The photocatalyst (2.0 mg) was dispersed in water/methanol solution (10 ml) by ultrasonication. Then, 50 μL DMPO (5,5-dimethyl-L-pyrroline N-oxide, 50 mM) was added in the suspension (200 μL) for DMPO-OH^•^ and DMPO-O_2_^•−^ measurements. The photocatalyst was added in the quartz tube and irradiated by UV light.

## Results and Discussion

### Compositional and Structural Characteristics of Mesoporous Sb_2_O_3_/TiO_2_ Heterojunctions

**FIGURE 1:**
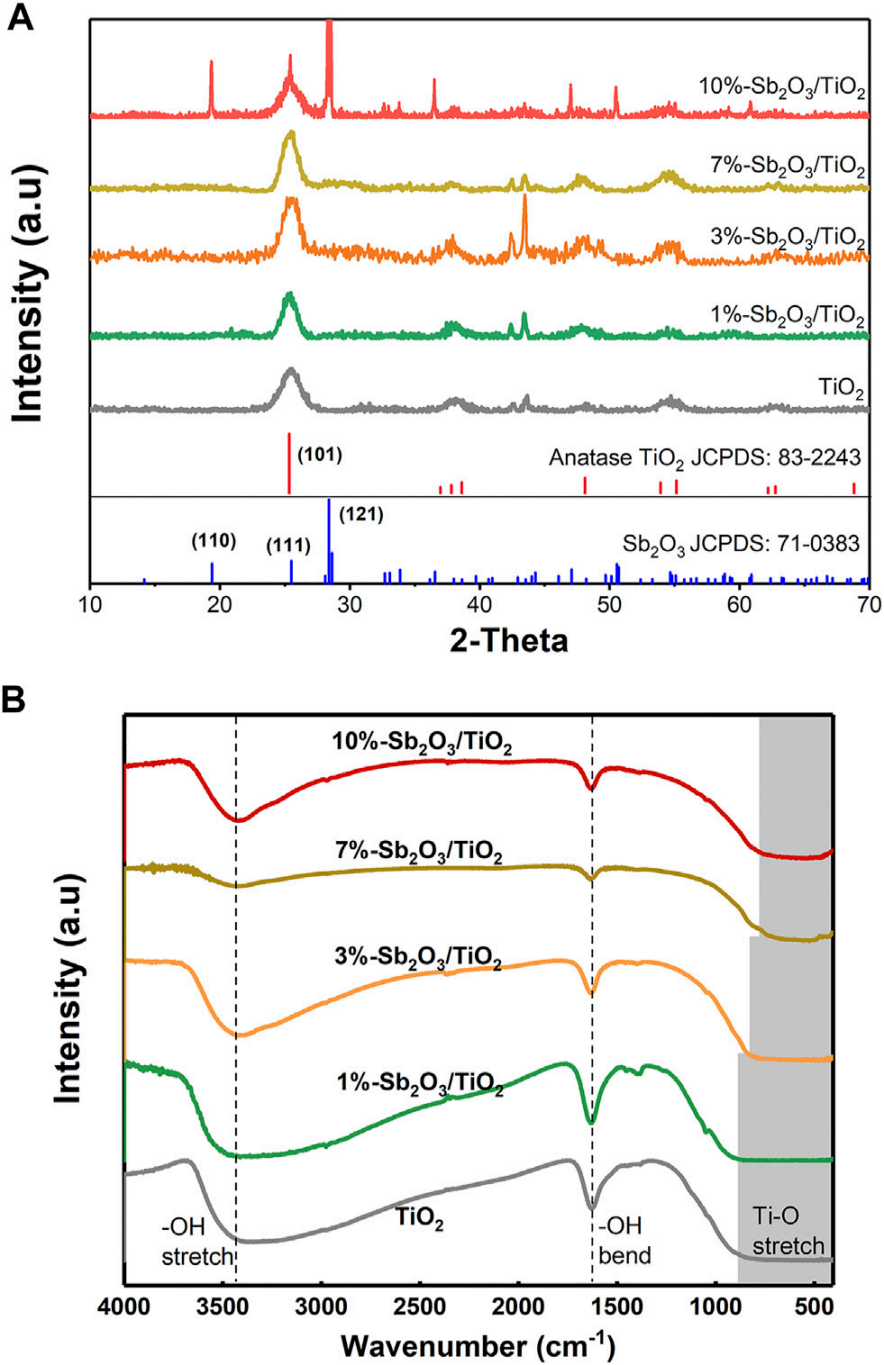
**(A)** XRD patterns and **(B)** FT-IR spectra of TiO_2_, 1%-Sb_2_O_3_/TiO_2_, 3%-Sb_2_O_3_/TiO_2_, 7%-Sb_2_O_3_/TiO_2_, and 10%-Sb_2_O_3_/TiO_2_.

As shown in Figure 1A, the XRD patterns of the as-prepared TiO_2_ and 1–10%-Sb_2_O_3_/TiO_2_ composite displayed a distinct diffraction peak at 2θ = 25.33°, which was indexed to the (101) plane of anatase TiO_2_ (JCPDS No. 83-2243). After loading Sb_2_O_3_, the two new peaks at 43.08 and 43.67° were assigned to the (220) and (240) planes of TiO (JCPDS No. 72-0020), which was probably attributed to the partial replacement of Ti-O by Sb-O. As the ratio of Sb_2_O_3_ to TiO_2_ increased, the intensity of the above two peaks first increased, then became most prominent in the pattern of 3%-Sb_2_O_3_/TiO_2_, and finally decayed. In the meantime, it was also noted that, the diffraction peaks of the (121) plane of Sb_2_O_3_ (JCPDS No. 71-0383) was only observed in 10%-Sb_2_O_3_/TiO_2_. Since the peak position for the (111) plane of Sb_2_O_3_ overlapped with the (121) plane of TiO_2_, the above phenomena suggested that, at a low loading content, the Sb_2_O_3_ nanoparticles were embedded in the mesoporous TiO_2_; and when the loading was high, the excess amount of Sb_2_O_3_ grow outside of the TiO_2_ framework, ending up as the higher energy crystal facets found in the 10%-Sb_2_O_3_/TiO_2_. The findings from XRD patterns were further supported by the FT-IR spectra of TiO_2_ and Sb_2_O_3_/TiO_2_ composites (Figure 1B). The wide finger band under 1,000 cm^−1^ was assigned to the grid structure of Ti-O-Ti. Hence, the narrowed the range of Ti-O-Ti peak, as the Sb_2_O_3_ content increased, also indicated the successful incorporation of Sb into TiO_2_ structure. Besides, the other two bands located at 1,636 and 3,438 cm^−1^ were normally ascribed to the bending and stretching of −OH.

**FIGURE 2:**
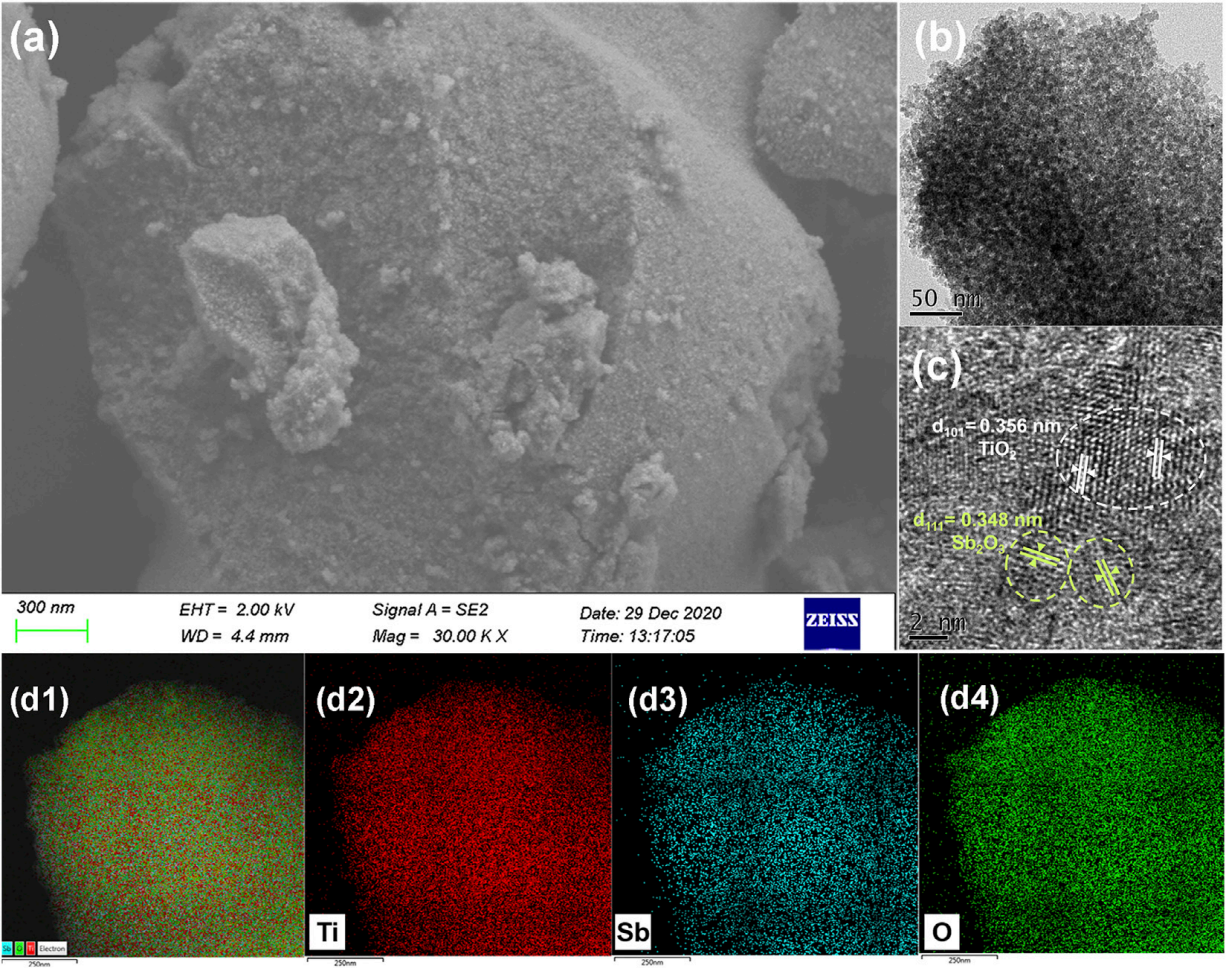
**(A)** SEM, **(B)** TEM, and **(C)** HRTEM images of 3%-Sb_2_O_3_/TiO_2_. **(D)** Elemental mapping of titanium, antimony, and oxygen from the same area with the same scale bar in the 3%-Sb_2_O_3_/TiO_2_.

Morphologies of the as-synthesized 3%-Sb_2_O_3_/TiO_2_ were further characterized by their SEM and TEM image Supplementary Figure S1. As seen in Figures 2A,B, the coarse bowl-like Sb_2_O_3_/TiO_2_ particles were composed of nanoparticles with a mean particle size of ca. 10 nm and interconnected pores in the similar range. The HRTEM image (Figure 2C) revealed that those nanoparticles were mainly of anatase TiO_2_ with a d-spacing of 0.356 nm for the (101) plane, surrounded by the Sb_2_O_3_ nanocrystals with a d-spacing of 0.348 nm for the (111) plane, which was in good accordance with the XRD results. In addition, the elemental mapping of Sb_2_O_3_/TiO_2_ (Figure 2D) also confirmed the uniform distribution of Sb (blue) and Ti (red) in the composites.

**FIGURE 3:**
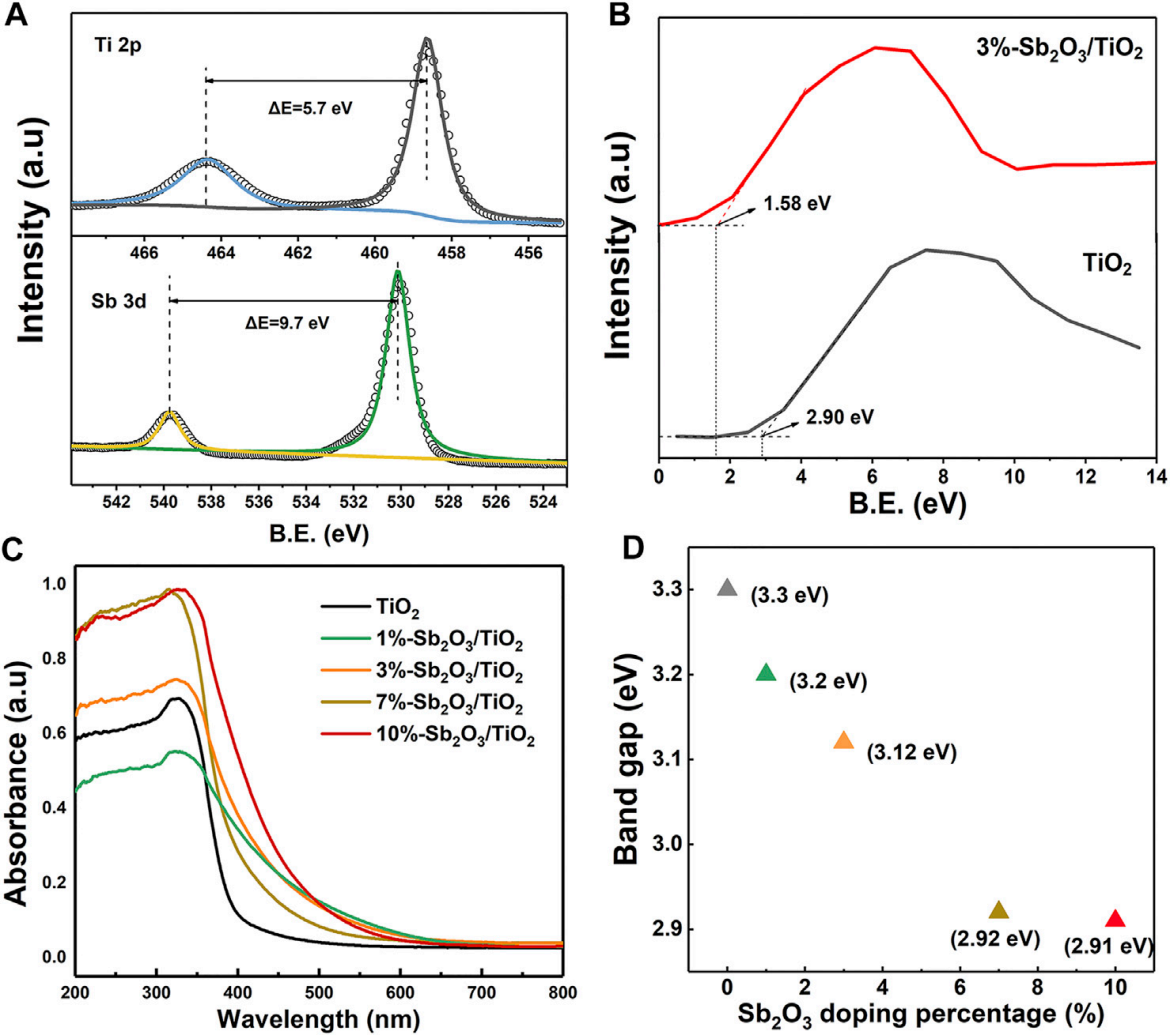
**(A)** XPS Ti 2p and Sb 3 days spectra of 3%-Sb_2_O_3_/TiO_2_. **(B)** XPS valence band spectra of TiO_2_ and 3%-Sb_2_O_3_/TiO_2_. **(C)** UV-vis spectra and **(D)** band gap of TiO_2_, 1%-Sb_2_O_3_/TiO_2_, 3%-Sb_2_O_3_/TiO_2_, 7%-Sb_2_O_3_/TiO_2_, and 10%-Sb_2_O_3_/TiO_2_.

As shown in the Ti 2p and Sb 3 days XPS spectra of 3%-Sb_2_O_3_/TiO_2_ (Figure 3A), the two peaks at 464.33 and 458.63 eV were assigned to the Ti 2p_1/2_ and Ti 2p_2/3_ peaks of anatase TiO_2_, respectively, with a characteristic splitting of 5.7 eV; and similarly, the other two at 539.96 and 530.26 eV were ascribed to the Sb 3d_3/2_ and Sb 3d_5/2_ peaks of Sb_2_O_3_. These observations were consistent with the aforementioned XRD and TEM measurements, affirming once again the formation of Sb_2_O_3_/TiO_2_ heterojunctions. The XPS valence band of TiO_2_ and 3%-Sb_2_O_3_/TiO_2_ was estimated to be at 2.90 and 1.58 eV (Figure 3B), respectively, proving that the integration of Sb_2_O_3_ was capable of raising the valence band to prevent the indirect OH^•^ pathway (the redox potential for H_2_O/OH^•^ was 2.27 eV). The light absorption characteristics of mesoporous TiO_2_ and Sb_2_O_3_/TiO_2_ with different Sb_2_O_3_ contents were demonstrated in Figure 3C. It was found that the light adsorption edge of mesoporous TiO_2_ located at ca. 400 nm and the corresponding band gap was calculated to be 3.30 eV, consistent with previous studies. In comparison, mesoporous Sb_2_O_3_/TiO_2_ heterojunctions exhibited a red shift in the absorption edge, which became ever more significant as the loading of Sb_2_O_3_ increased. The band gap energies of the mesoporous TiO_2_ and the Sb_2_O_3_/TiO_2_ composites were estimated based on the intercept of the Tauc plot of (αhν)^2^ vs. the photon energy (hv), which decreased from 3.30–2.91 eV as the content of Sb_2_O_3_ increased from 0–10% (Figure 3D). Hence, lower excitation energy was required to initiate the electron transition in Sb_2_O_3_/TiO_2_. Meanwhile, the broader range of light adsorption and higher intensity by the heterojunctions allowed the utilization of more irradiation.

The N_2_ adsorption-desorption isotherms were adopted to characterize the porous structures of TiO_2_ and Sb_2_O_3_/TiO_2_ composites. It was seen in Supplementary Figure S1 that all these samples exhibited a typical type IV curves with H2 hysteresis loops, which corresponded to a well-defined mesoporous structure. The narrow pore size distributions (derived from the adsorption branches using BJH model) indicated that the resulting mesopores were quite uniform in each sample. It was also noted that the specific surface area of Sb_2_O_3_/TiO_2_ was slightly smaller than that of pristine TiO_2_, and kept decreasing as the Sb_2_O_3_ loading increased, which was probably attributed to the partial pore blocking by nanoconfined Sb_2_O_3_. The corresponding pore characterization data were summarized in Supplementary Table S1.

### Photocatalytic PFOA Degradation Activities and Mechanisms of Sb_2_O_3_/TiO_2_ Heterojunctions

**FIGURE 4:**
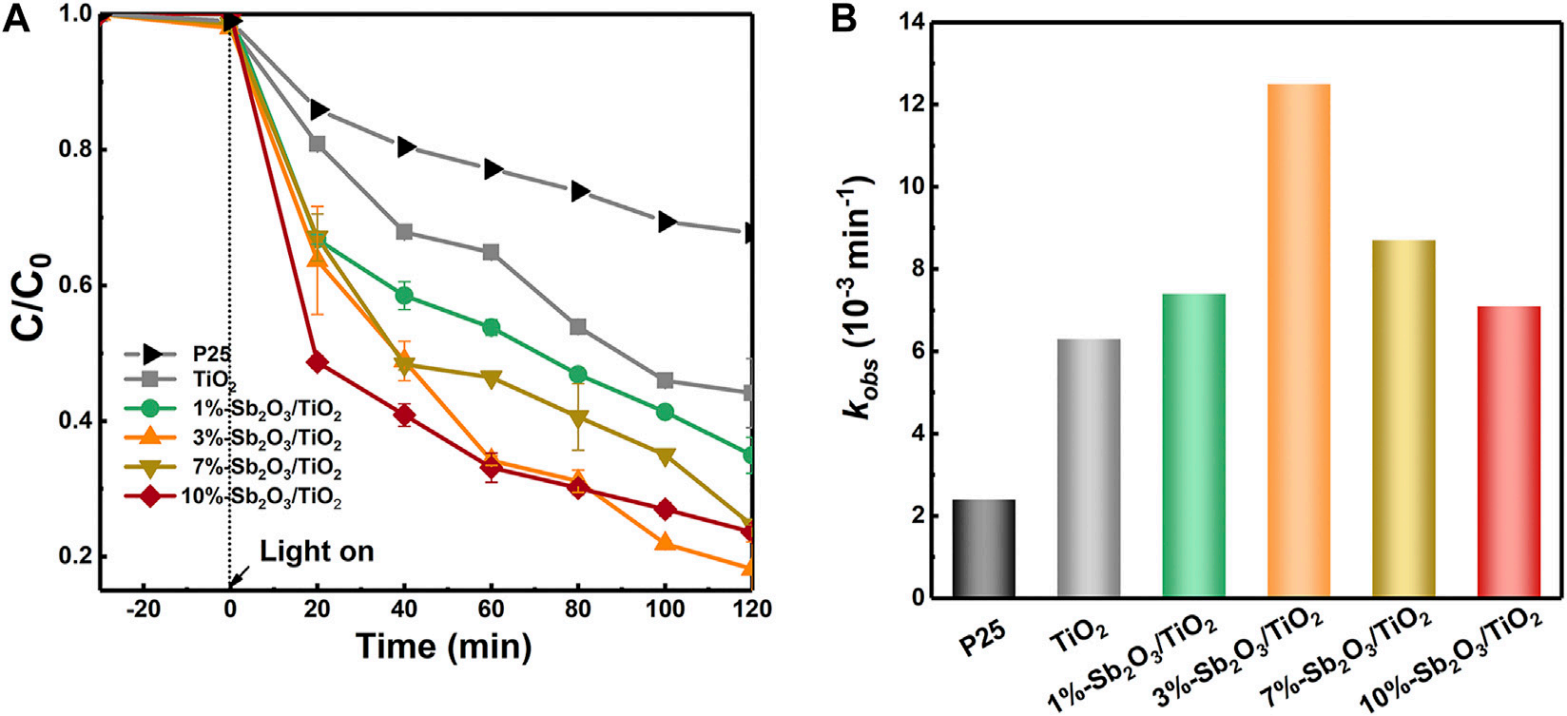
**(A)** PFOA degradation performance and **(B)** rate constants of P25, TiO_2_, 1%-Sb_2_O_3_/TiO_2_, 3%-Sb_2_O_3_/TiO_2_, 7%-Sb_2_O_3_/TiO_2_, and 10%-Sb_2_O_3_/TiO_2_.

Figure 4A presented the performance of photocatalytic degradation of PFOA by TiO_2_ embedded with various amount of Sb_2_O_3_. When the initial PFOA concentration was 10 ppm and catalyst dosage was 0.25 g/L, the as-synthesized mesoporous TiO_2_ exhibited a 73% higher removal rate (55.9%) than the commercial P25 (32.3%) after 120 min of operation. The nanoconfinement of Sb_2_O_3_ into mesoporous TiO_2_ framework further enhanced the PFOA degradation efficiency, and a maximal removal rate of 81.83% was realized by 3%-Sb_2_O_3_/TiO_2_. Contribution of the PFOA adsorption by catalysts should be negligible—before light was turned on, the solution had already been stirred in the dark for 30 min and no apparent decrease of PFOA concentration was observed. The PFOA removal rates between 20 and 120 min were used to calculate the degradation kinetics. According to the Langmuir-Hinshelwood model, a pseudo-first-order kinetics was applied to fit the photo-induced rate constant. Based on that, the degradation kinetics of mesoporous TiO_2_ (6.3 × 10^−3^ min^−1^) and 3%-Sb_2_O_3_/TiO_2_ (12.6 × 10^−3^ min^−1^) were found to be 1.6- or 4.2-times higher than that of the commercial P25 (6.3 × 10^–3^ min^−1^). More importantly, a volcano-type relationship was seen between the Sb_2_O_3_ loading content and the photocatalytic PFOA degradation efficiency (Figure 4B).

**FIGURE 5:**
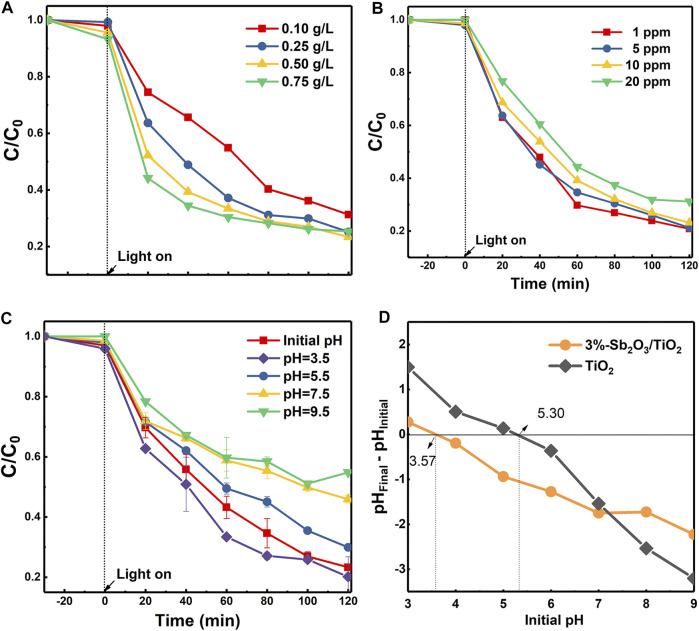
The influence of **(A)** catalyst dosage, **(B)** initial PFOA concentration, and **(C)** solution pH on the photocatalytic degradation of PFOA by 3%-Sb_2_O_3_/TiO_2_, and **(D)** the point of zero charge of TiO_2_ and 3%-Sb_2_O_3_/TiO_2_.

The resulting 3%-Sb_2_O_3_/TiO_2_ with an optimal activity were selected for the following examination of operating conditions. It was displayed in Figure 5A that, as the dosage of 3%-Sb_2_O_3_/TiO_2_ catalysts increased, the initial removal kinetics within the first 20 min was greatly enhanced, probably due to the larger total amount of active sites for PFOA degradation. On the other hand, despite the fact that the final degradation rate at 120 min was higher in the 0.25 g/L test than in the 0.1 g/L one, further addition of 3%-Sb_2_O_3_/TiO_2_ improved little the overall performance, because the excess catalysts may interfere the light transmission or cause agglomeration of the catalysts, resulting in the scattering of the irradiated light and reduction of photon utilization. The impact of initial PFOA concentration was examined as well (Figure 5B). When 1 or 5 ppm PFOA was added, the two degradation curves were almost identical; and if the concentration of PFOA went even higher, the overall removal rate would drop a little, but a nearly 70% degradation at 120 min was still achievable for 20 ppm PFOA. The pH value of the solution also played a crucial role in the photocatalytic PFOA degradation performance. Note that the initial pH of the solution was ca. 4.4, a negative correlation was identified between the PFOA degradation activity and the pH value (Figure 5C). One possible reason for this phenomenon should be attributed to the relationship between the acid dissociation constant (p*K*_a_) of PFOA and the point of zero charge (PZC) of photocatalysts. The p*K*_a_ value of PFOA was reported to be 2.8 (Goss, 2008), and it was shown in Figure 5D that the PZC of TiO_2_ and 3%-Sb_2_O_3_/TiO_2_ were 5.30 and 3.57, respectively. Therefore, when the pH of solution was higher than the p*K*_a_ of PFOA and the PZC of TiO_2_ and Sb_2_O_3_/TiO_2_, both the PFOA and the photocatalysts were predominantly negatively charged. Such a repelling effect became even more significant as the pH value increased, which explained the lower degradation activity at a higher pH.

**FIGURE 6:**
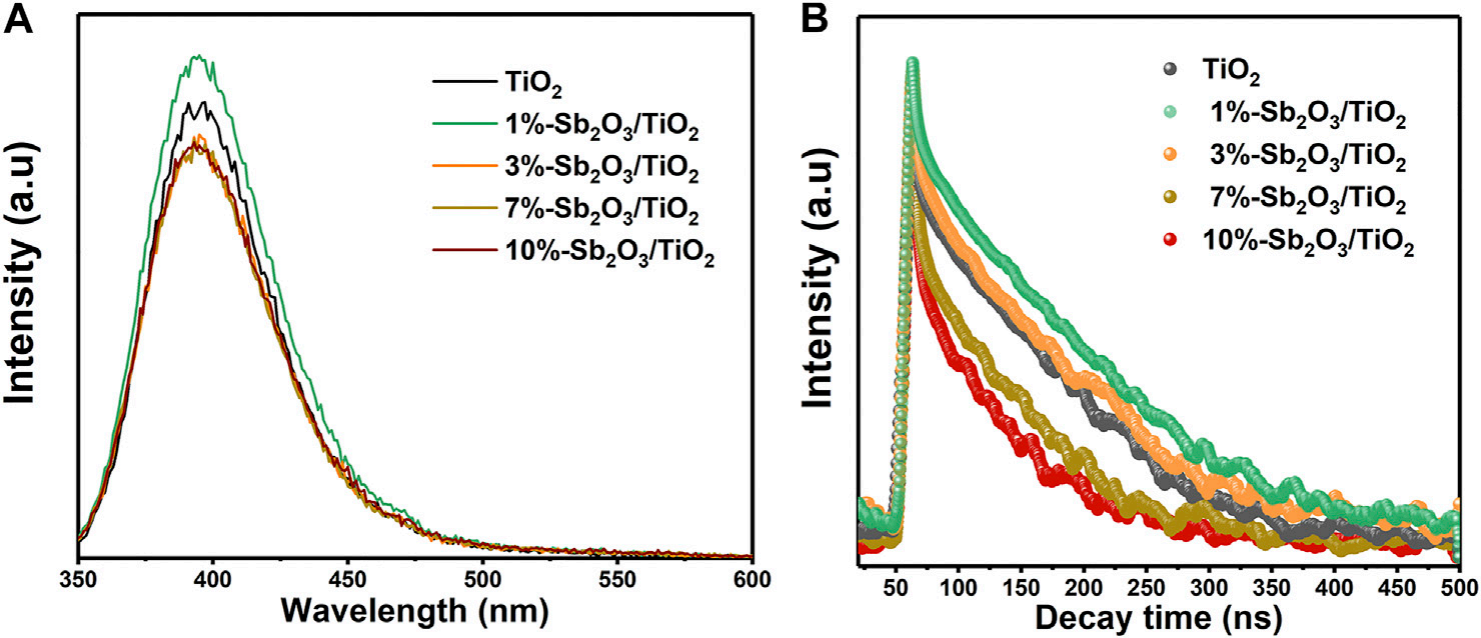
**(A)** PL emission spectra, and **(B)** TR-PL spectra of TiO_2_, 1%-Sb_2_O_3_/TiO_2_, 3%-Sb_2_O_3_/TiO_2_, 7%-Sb_2_O_3_/TiO_2_, and 10%-Sb_2_O_3_/TiO_2_.

Photoluminescence spectroscopy (PL) and time-resolved photoluminescence spectroscopy (TR-PL) were then adopted to examine the behavior of electron-hole recombination. As seen in Figure 6A, all the samples exhibited an emission band from 350 to 500 nm in the PL spectra measured at an excitation wavelength of 325 nm. The lower emission intensity generally corresponded to a slower recombination rate, and it was found that the emission intensity of TiO_2_, 1 and 3%-Sb_2_O_3_/TiO_2_ was close to each other, while those of 7 and 10%-Sb_2_O_3_/TiO_2_ were higher. The average lifetime of the emission decay, calculated from the TR-PL spectra (Figure 6B), was 27.09, 33.28, 26.26, 14.98, and 9.54 ns, for the pristine TiO_2_ and 1, 3, 7 and 10% Sb_2_O_3_/TiO_2_ heterojunctions, in accordance with the finding in PL intensity. In short, compared with the pristine TiO_2_, the electron-hole recombination of 1%-Sb_2_O_3_/TiO_2_ was slightly slower, the 3%- one was comparable, and the 7 and 10%- ones were faster.

**FIGURE 7:**
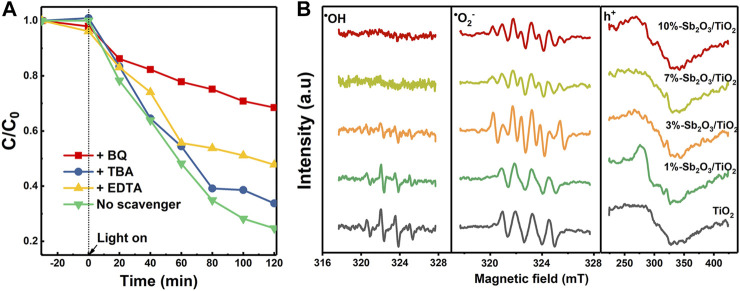
**(A)** The effects of various scavengers on the PFOA degradation kinetics of 3%-Sb_2_O_3_/TiO_2_, and **(B)** ESR spectra under UV light for OH^•^, O_2_^•−^, and h^+^ intermediates.

To elucidate the origin of the enhanced photocatalytic PFOA degradation activity by Sb_2_O_3_/TiO_2_, a series of scavengers, including t-butanol to quench OH^•^, p-benzoquinone (BQ) to quench O_2_^•−^, and EDTA as the scavenger for the h^+^, were employed to determine the specific role of each reactive species. As shown in Figure 7A, the degradation of PFOA was most significantly suppressed by BQ, then by EDTA, and almost negligible with TBA, suggesting that the photo-induced generation of O^2•−^and h^+^, rather than OH^•^, made the main contribution to the degradation of PFOA. Besides, the sum of the individual loss of photocatalytic activity by BQ and EDTA scavengers was almost equal to the total activity of 3%-Sb_2_O_3_/TiO_2_. This finding was in stark contrast to most of the previous studies on TiO_2_ based photocatalysts, but was highly consistent with the other characterization results in this work, e.g., the XPS valence band position and the band gap estimated from UV-vis spectra. The electron spin resonance (ESR) measurements were further conducted to resolve the aforementioned radicals, using DMPO as the spin-trap reagent (Figure 7B). The signal of DMPO-OH^•^ adduct was strong in the measurement with pristine TiO_2_, then gradually decayed in 1- and 3%-Sb_2_O_3_/TiO_2_, and became completely invisible when the Sb_2_O_3_ content reached 7% or higher. By contrast, only four peaks were observed in the O_2_^•−^ measurements with pristine TiO_2_ and 1%-Sb_2_O_3_/TiO_2_, but they were then splitted into eight when the Sb_2_O_3_ went up to 3% or higher, which was probably due to the lower PZC of the catalysts than the pH of the solution. The highest intensity of DMPO-O_2_^•−^ adduct in 3%-Sb_2_O_3_/TiO_2_ is a strong evidence to support the O_2_^•−^ dominant pathway proposed in this work for the photocatalytic degradation of PFOA.

**FIGURE 8:**
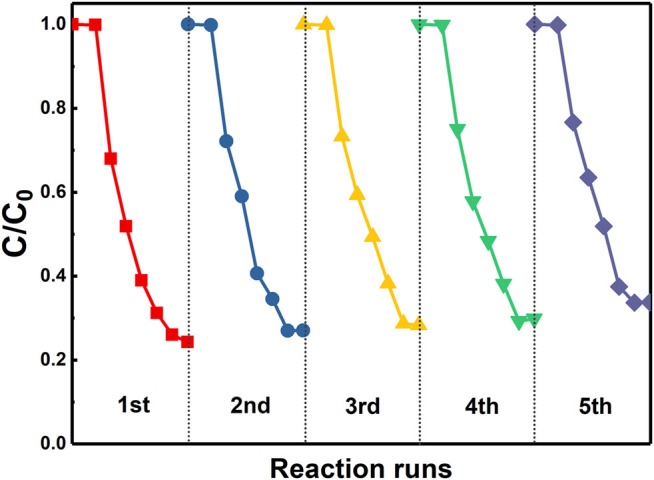
The PFOA degradation performance of 3%-Sb_2_O_3_/TiO_2_ in five consecutive cycles.

Finally, the reusability of the synthesized 3%-Sb_2_O_3_/TiO_2_ photocatalysts was investigated. After each cycle, the photocatalysts were recovered through the following steps: the suspension was first left for a while to allow the precipitation of the photocatalysts, the supernatant was then extracted and removed, the remaining photocatalysts were washed with DI water and ethanol for three times, and finally kept in the oven at 60°C for drying. The exact same reactor and photocatalysts were applied in the next cycle of measurement. The result of the recycling test was given in Figure 8. After five consecutive cycles of operation, ca. 88% of the initial photocatalytic degradation efficiency was retained, which clearly demonstrated the potential feasibility of using this high-performance photocatalyst in the long-term PFOA removal applications.

## Conclusion

**FIGURE 9:**
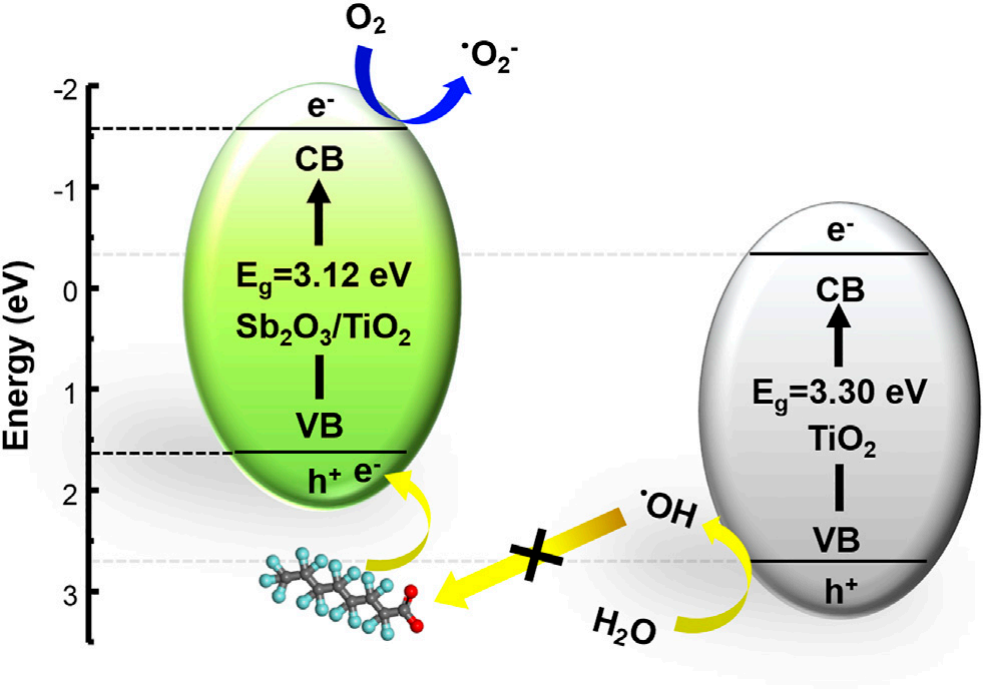
The schematic illustration for the enhanced photocatalytic PFOA degradation pathway by the 3%-Sb_2_O_3_/TiO_2_ heterojunctions.

In this study, a highly efficient mesoporous Sb_2_O_3_/TiO_2_ heterojunction was designed to enhance the photocatalytic activity of PFOA degradation. The embedding of Sb_2_O_3_ nanocrystals into mesoporous TiO_2_ framework was realized via a facile hydrothermal method. The crystal structures, morphology, chemical composition, and optical properties of the proposed Sb_2_O_3_/TiO_2_ catalysts were carefully examined. Most importantly, it was found that the valence band edge was raised, the band gap was reduced, and the light adsorption was enhanced. The resulting 3%-Sb_2_O_3_/TiO_2_ managed to remove 81.7% of the initial PFOA in 120 min, with a degradation rate 4.2 times faster than the commercial P25. Detailed mechanistic analysis revealed that the photo-generated superoxide radicals and holes were the two main contributor to the improved performance (Figure 9), while the photo-induced formation of hydroxyl radicals was prohibited, and the recombination of electron and hole remained the same. In addition, it was also noted that nearly 90% of the catalytic activity was successfully retained after five cycling tests, indicating the promise of this photocatalyst in practical applications.

## Data Availability

The original contributions presented in the study are included in the article/Supplementary Material, further inquiries can be directed to the corresponding author.
