# Supplementary material for: Enhanced Photocatalytic Degradation of Perfluorooctanoic Acid by Mesoporous Sb2O3/TiO2 Heterojunctions
Source: Front Chem. 2021 May 19;9:690520. doi: 10.3389/fchem.2021.690520 (PMC8170081; doi:10.3389/fchem.2021.690520)
Supplement: Supplementary file 1 [file DataSheet1.docx]

Supplementary Material







**(a1)**

**(a2)**

**(b2)**

**(b1)**







**(c2)**

**(c1)**












**(d1)**

**(d2)**

**(e2)**

**(e1)**







**Supplementary Figure 1**. N_2_ adsorption-desorption isotherms and pore distribution of (a) TiO_2_, (b) 1%-Sb_2_O_3_/TiO_2_, (c) 3%-Sb_2_O_3_/TiO_2_, (d) 7%-Sb_2_O_3_/TiO_2_, (e) 10%-Sb_2_O_3_/TiO_2_.


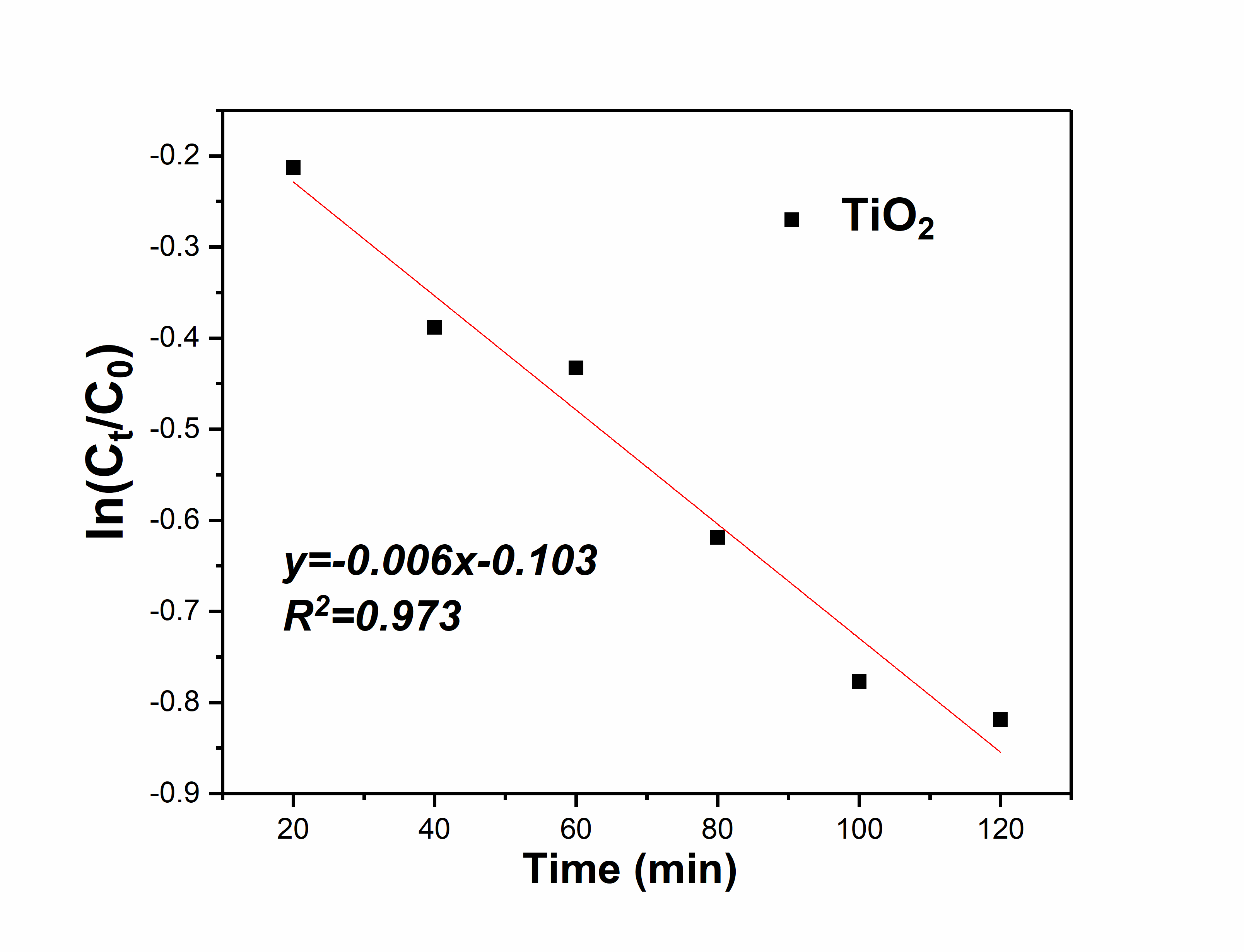

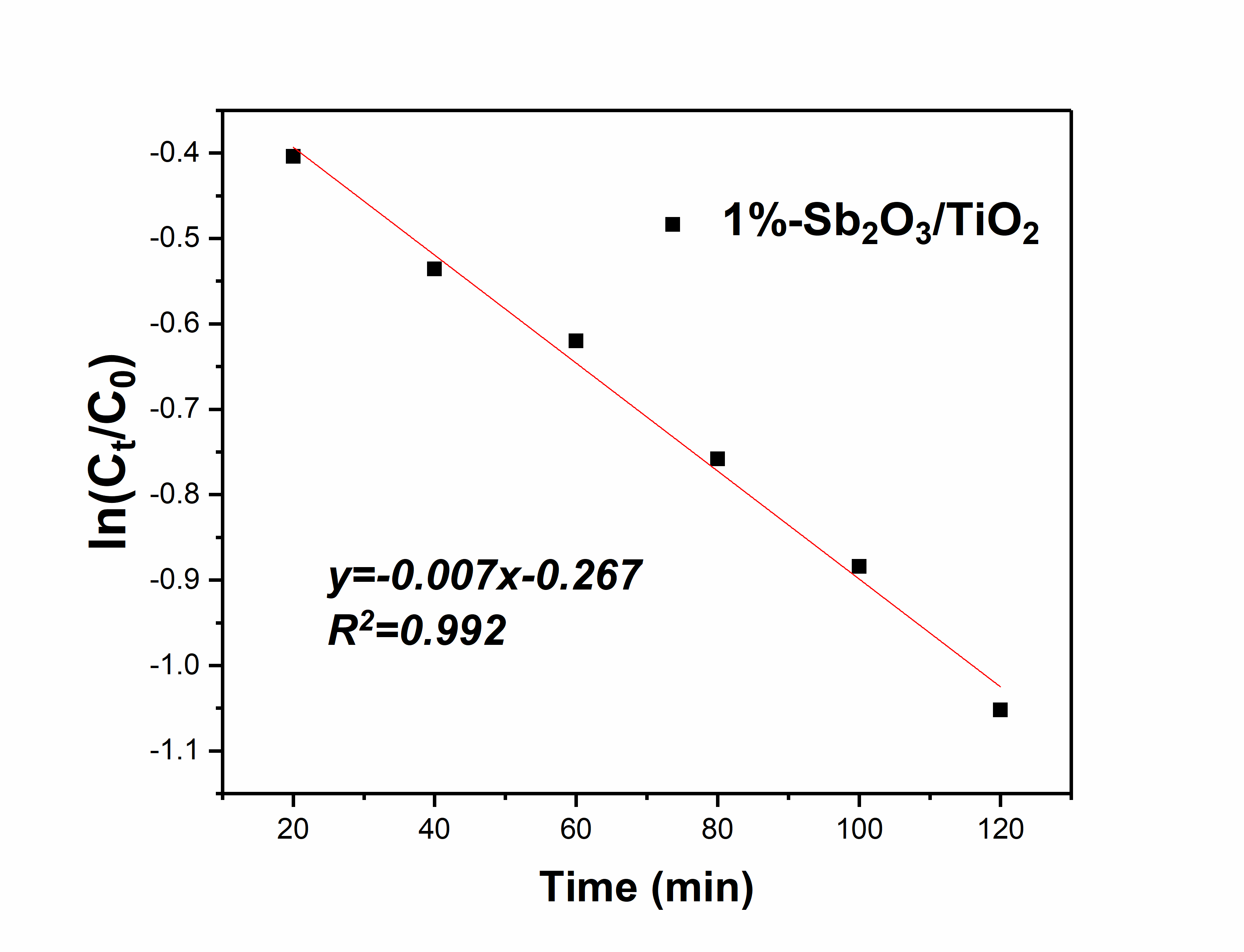


**(b)**

**(a)**


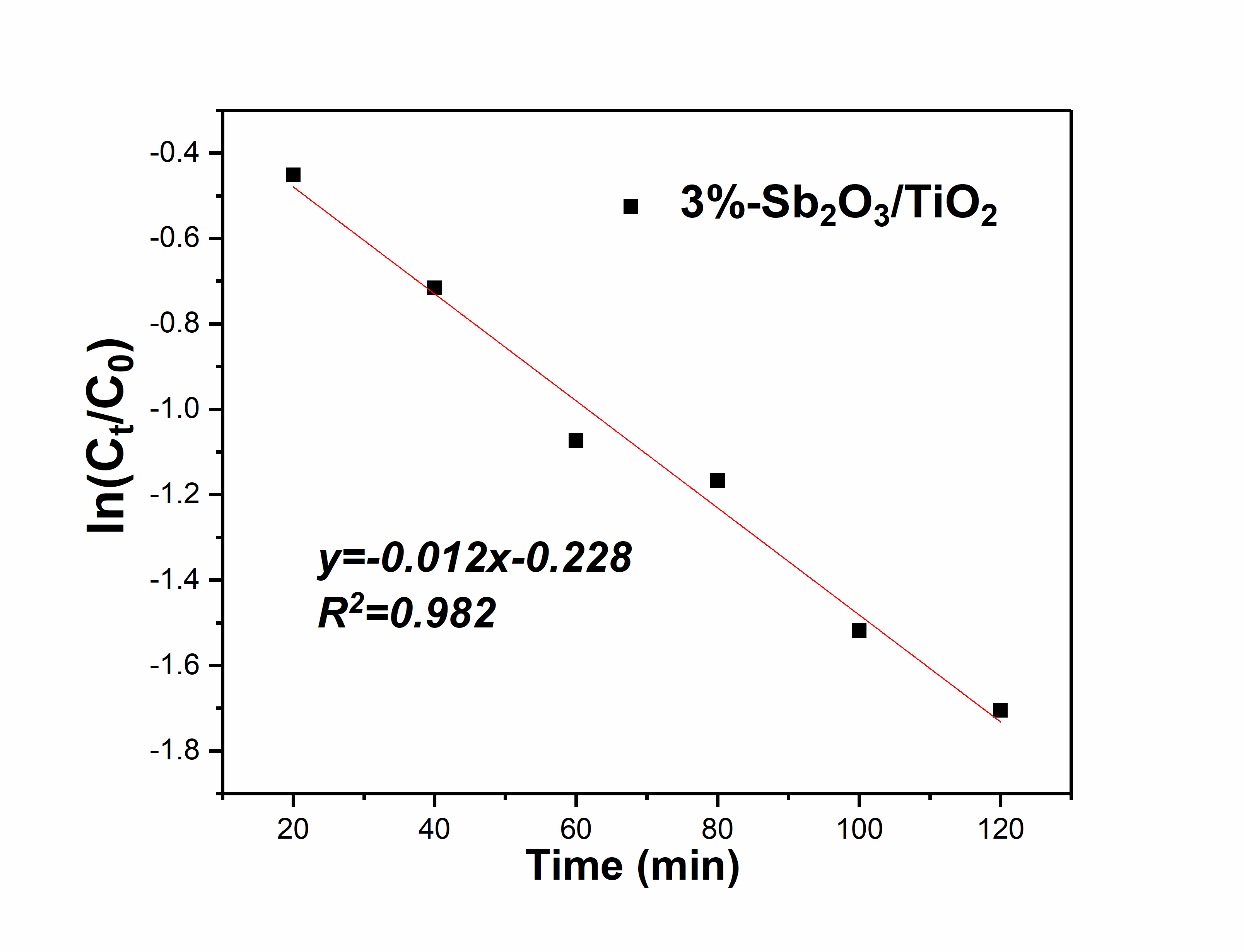

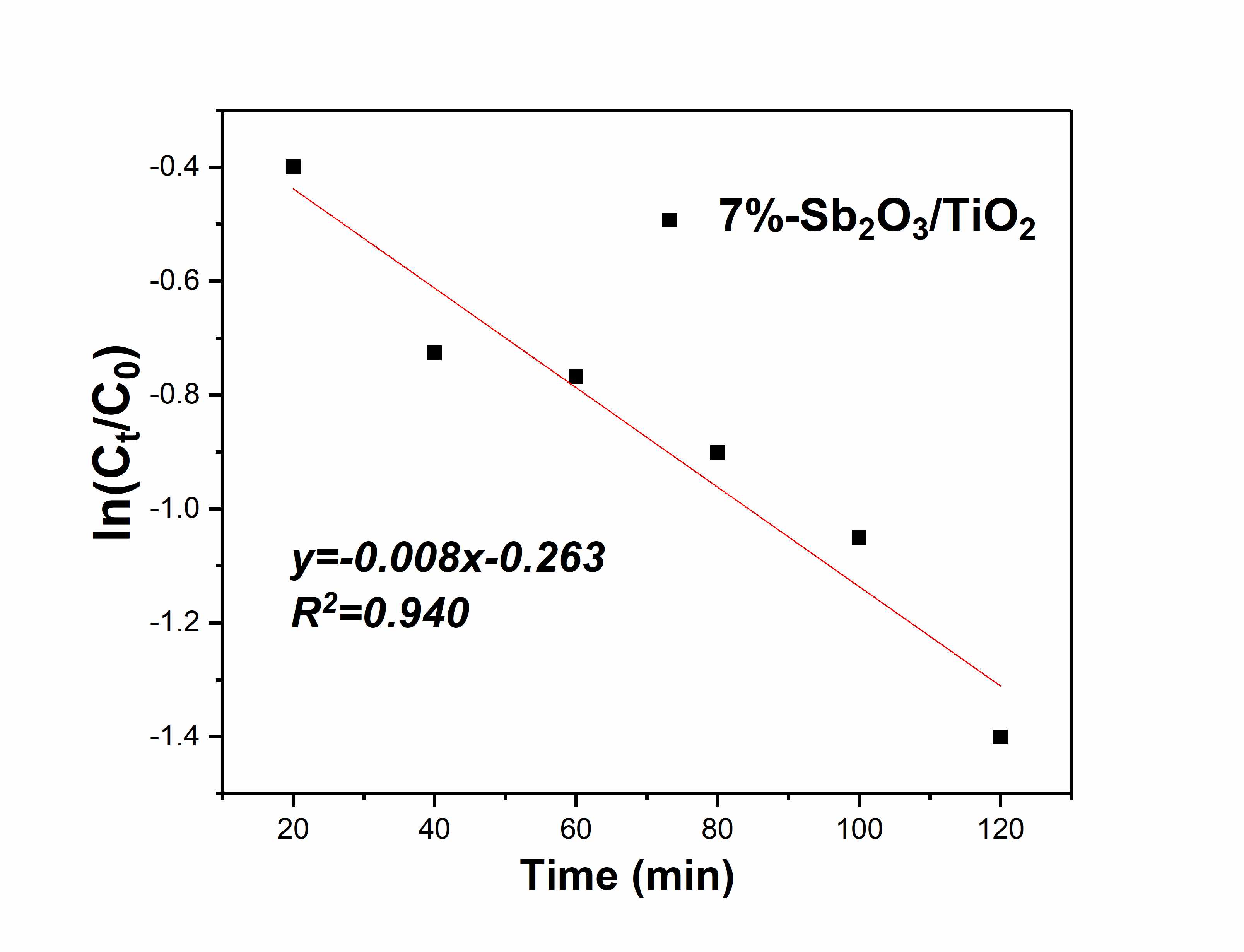


**(d)**

**(c)**


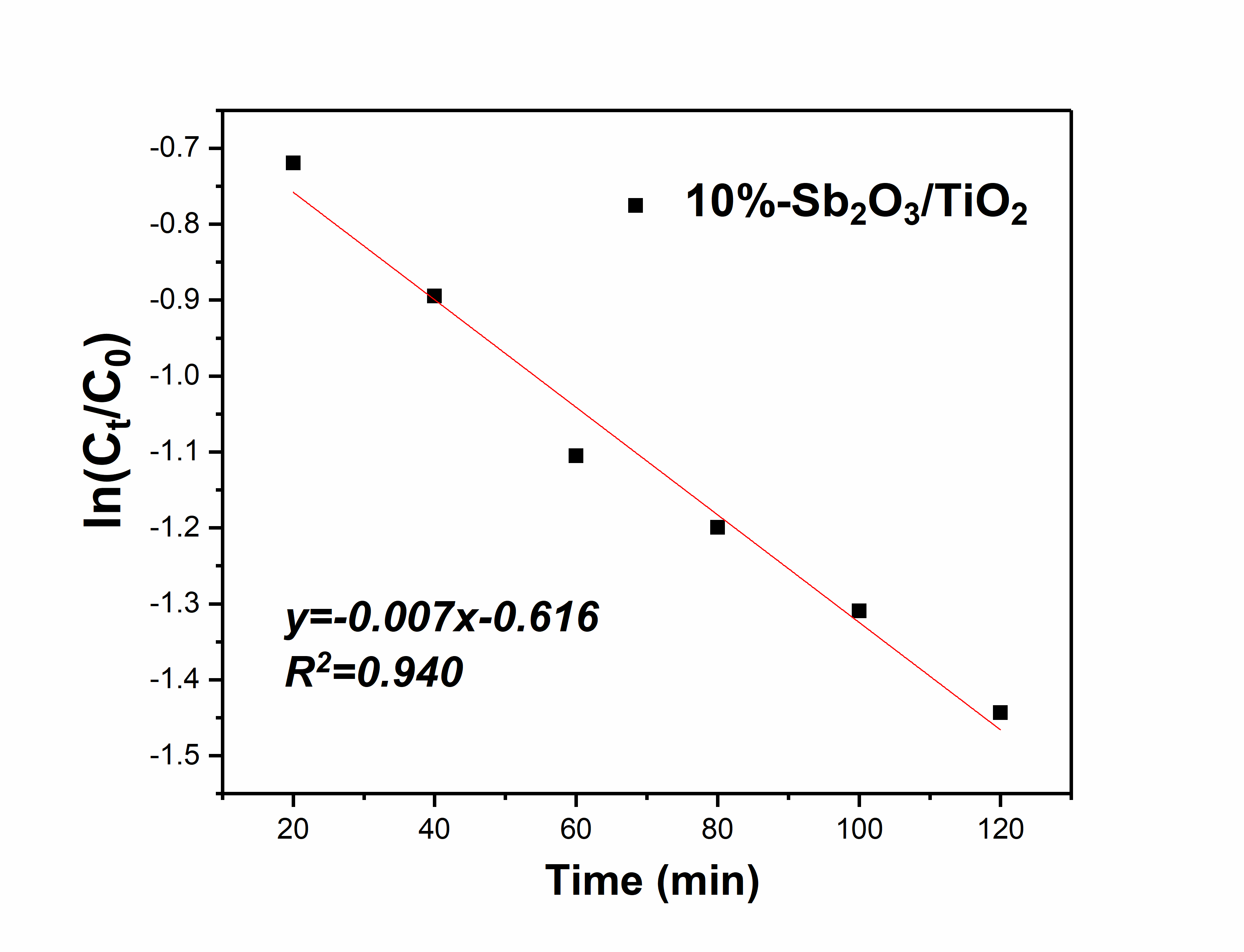


**(e)**

**Supplementary Figure 2**. The linear fitting results of kinetics of (a) TiO_2_, (b) 1%-Sb_2_O_3_/TiO_2_, (c) 3%-Sb_2_O_3_/TiO_2_, (d) 7%-Sb_2_O_3_/TiO_2_, (e) 10%-Sb_2_O_3_/TiO_2_.

**Table S1.** Summary of the pore characterization data of the samples.

| Sample | S_BET_^a^ | V_total_^b^ | Pore radius^c^ |
| --- | --- | --- | --- |
| TiO_2_ | 258.5 | 0.27 | 19.26 |
| 1%-Sb_2_O_3_/TiO_2_ | 218.5 | 0.33 | 25.33 |
| 3%-Sb_2_O_3_/TiO_2_ | 178.7 | 0.29 | 27.53 |
| 7%-Sb_2_O_3_/TiO_2_ | 180.9 | 0.25 | 23.47 |
| 10%-Sb_2_O_3_/TiO_2_ | 205.0 | 0.19 | 18.27 |

^a^ Specific surface area (m^2^ g^-1^);

^b^ Total volume of pores (cm^3^ g^-1^);

^c^ Average pore width (nm)

**Table S2**. The kinetics parameters for PFOA degradation in the photocatalytic system.

| Parameters | |  | Degradation efficiency (%) |  | Rate constant  (min^-1^) |  | R^2^ |
| --- | --- | --- | --- | --- | --- | --- | --- |
| Sb_2_O_3_ content of Sb_2_O_3_/TiO_2_ | 0% |  | 55.9 |  | 0.0063 |  | 0.992 |
|  | 1% |  | 56.9 |  | 0.0073 |  | 0.985 |
|  | 3% |  | 81.7 |  | 0.0125 |  | 0.940 |
|  | 7% |  | 75.4 |  | 0.0087 |  | 0.981 |
|  | 10% |  | 76.4 |  | 0.0071 |  | 0.974 |
|  |  |  |  |  |  |  |  |
| Catalyst dosage | 0.10 g/L |  | 70.8 |  | 0.0106 |  | 0.994 |
|  | 0.25 g/L |  | 74.9 |  | 0.0127 |  | 0.967 |
|  | 0.50 g/L |  | 76.7 |  | 0.0109 |  | 0.969 |
|  | 0.75 g/L |  | 74.7 |  | 0.0092 |  | 0.905 |
|  |  |  |  |  |  |  |  |
| PFOA concentration | 1 ppm |  | 79.2 |  | 0.011 |  | 0.921 |
|  | 5 ppm |  | 78.8 |  | 0.0104 |  | 0.971 |
|  | 10 ppm |  | 76.8 |  | 0.011 |  | 0.982 |
|  | 20 ppm |  | 68.8 |  | 0.0092 |  | 0.940 |
|  |  |  |  |  |  |  |  |
| pH | Initial |  | 79.9 |  | 0.0113 |  | 0.955 |
|  | 3.5 |  | 76.7 |  | 0.0113 |  | 0.995 |
|  | 5.5 |  | 70.1 |  | 0.0088 |  | 0.992 |
|  | 7.5 |  | 54.1 |  | 0.0045 |  | 0.995 |
|  | 9.5 |  | 45.2 |  | 0.0038 |  | 0.847 |
|  |  |  |  |  |  |  |  |
| Scavenger | No |  | 75.3 |  | 0.0122 |  | 0.987 |
|  | BQ |  | 31.6 |  | 0.0023 |  | 0.995 |
|  | TBA |  | 66.3 |  | 0.0092 |  | 0.951 |
|  | EDTA |  | 52.3 |  | 0.0056 |  | 0.892 |
|  |  |  |  |  |  |  |  |
| Reaction runs | 1^st^ |  | 75.7 |  | 0.0106 |  | 0.967 |
|  | 2^nd^ |  | 73.0 |  | 0.0106 |  | 0.948 |
|  | 3^rd^ |  | 71.7 |  | 0.0103 |  | 0.973 |
|  | 4^th^ |  | 70.2 |  | 0.0098 |  | 0.961 |
|  | 5^th^ |  | 66.3 |  | 0.009 |  | 0.935 |

**Table S3**. The lifetime of synthesized photocatalysts.

| Sample | τ_1_ (ns) | Rel (%) | τ_2_ (ns) | Rel (%) | τ_average_ (ns) |
| --- | --- | --- | --- | --- | --- |
| TiO_2_ | 1.26 | 41.01 | 45.04 | 58.99 | 27.086 |
| 1%-Sb_2_O_3_/TiO_2_ | 1.55 | 30.54 | 47.24 | 69.46 | 33.286 |
| 3%-Sb_2_O_3_/TiO_2_ | 1.73 | 42.68 | 44.53 | 57.32 | 26.263 |
| 7%-Sb_2_O_3_/TiO_2_ | 1.37 | 63.69 | 36.83 | 38.31 | 14.982 |
| 10%-Sb_2_O_3_/TiO_2_ | 1.42 | 74.06 | 32.74 | 25.94 | 9.544 |
